# Social cohesion matters in health

**DOI:** 10.1186/1475-9276-12-87

**Published:** 2013-10-28

**Authors:** Ying-Chih Chuang, Kun-Yang Chuang, Tzu-Hsuan Yang

**Affiliations:** 1 School of Public Health, Taipei Medical University, 250 Wu-Hsing St., Taipei City 110, Taiwan

**Keywords:** Social cohesion, Self-rated health, Liberty, Equality, Solidarity, Social capital

## Abstract

**Introduction:**

The concept of social cohesion has invoked debate due to the vagueness of its definition and the limitations of current measurements. This paper attempts to examine the concept of social cohesion, develop measurements, and investigate the relationship between social cohesion and individual health.

**Methods:**

This study used a multilevel study design. The individual-level samples from 29 high-income countries were obtained from the 2000 World Value Survey (WVS) and the 2002 European Value Survey. National-level social cohesion statistics were obtained from Organization of Economic Cooperation and Development datasets, World Development Indicators, and Asian Development Bank key indicators for the year 2000, and from aggregating responses from the WVS. In total 47,923 individuals were included in this study. The factor analysis was applied to identify dimensions of social cohesion, which were used as entities in the cluster analysis to generate a regime typology of social cohesion. Then, multilevel regression models were applied to assess the influences of social cohesion on an individual’s self-rated health.

**Results and discussion:**

Factor analysis identified five dimensions of social cohesion: social equality, social inclusion, social development, social capital, and social diversity. Then, the cluster analysis revealed five regimes of social cohesion. A multi-level analysis showed that respondents in countries with higher social inclusion, social capital, and social diversity were more likely to report good health above and beyond individual-level characteristics.

**Conclusions:**

This study is an innovative effort to incorporate different aspects of social cohesion. This study suggests that social cohesion was associated with individual self-rated after controlling individual characteristics. To achieve further advancement in population health, developed countries should consider policies that would foster a society with a high level of social inclusion, social capital, and social diversity. Future research could focus on identifying possible pathways by which social cohesion influences various health outcomes.

## Introduction

The relationship between social cohesion and population health has intrigued many researchers in the past two decades [1], and many policymakers regard social cohesion as a solution to the increasing health inequality and decline of civil culture [2]. Nevertheless, social cohesion has invoked debate due to the vagueness of its definition and an inability of current measurements to capture the full meaning of the concept [3]. Green and colleagues referred to social cohesion as “the property by which the whole society, and individuals within, are bound together through the action of specific attitudes, behaviors, rules, and institutions, which rely on consensus rather than pure coercion” [4]. In this definition, attitudes and behaviors of citizens and governments, together with institutional and structural features, foster a consensus by society. However, most recent research measured social cohesion by way of trust and association participation [5]. The problem with this approach is that only those limited aspects of attitudinal and behavioral measurements are covered, and thus, it is indistinguishable from the concept of social capital. Moreover, measurements, such as institutional features (i.e., a welfare state) and attitudes toward social exclusion, were not considered [6]. To further clarify the concept of social cohesion, this paper used indicators from several global datasets and attempted to reexamine and develop the measurements of social cohesion.

Prior studies have regarded social cohesion as an important determinant of population health. With widening income inequality in worldwide, researchers and policy makers were concerned about the negative impact of income inequality on social trust and community structure (i.e., public education and social welfare program), which may further worsen population health [7]. To address the extent of influence of social cohesion on individual health, this study used a multilevel study design to analyze the relationship between social cohesion and individual health above and beyond individual characteristics.

### From social capital to social cohesion

Many prior studies, particularly in the field of health research, used the concepts of social capital and social cohesion interchangeably. Social capital was typically defined as resources imbedded in social networks such as norms and trust that can facilitate coordination and cooperation for people to achieve interests [8-10]. Some researchers have suggested that although there are some broad similarities between social capital and social cohesion, they operate at different levels. Researchers generally agree that social capital, with its emphasis on norms derived from networks, has its foundations in groups and communities [11]. Social cohesion, on the other hand, usually refers to cohesion at a societal level, which is normally taken to be at the level of a nation or state [6,12]. Some other researchers have examined the relationship between social capital and social cohesion [13,14]. They regarded social capital as an aggregation comprising three stages, with each stage building on the one that came before. In the first stage, social capital describes trust and social participation among face-to-face, horizontal networks like personal contacts with neighbors and friends. In the second stage, social capital further includes larger communities and is not restricted to face-to-face networks. The third stage is where social capital meets social cohesion, which includes trust, networks at the societal level, plus connections to formal and institutionalized power in a society. The above integration is also similar to the categorization of social capital proposed by Szreter and Woolcok [15], which separate social capital into three stages of bonding, bridging, and linking forms of social capital.

### Integrated models of social cohesion

To construct social cohesion as a macro- and societal-level concept, Bernard proposed an integrated model of democratic dialectic, which combines social and relational characteristics and institutional characteristics [3]. The model contains three principles (liberty, equality, and solidarity) that interact in complex dialectical relationships, and a cohesive society can only exist when the three principles reach a good balance [16]. For instance, a society without liberty is in danger of coercion and enslavement; however, a society that predominantly emphasizes liberty, in particular economic liberty, can lead to polarization and social dislocation. Countries that emphasize liberty and equality, but not solidarity, may have government policies and regulations to provide basic state-run health welfare and educational services, but the economic inequalities fostered by entrepreneurial liberty may enable the rich to get even better provisions than others. This model is referred to as “inclusive democracy”. Under this context, government efforts are usually too uniform and private services are too expensive. Therefore, it is the call to solidarity, such as by community sectors, through which resources are organized and services provided to people in need [3].

Berger-Schmitt and Noll took Bernard’s model one step further and provided a useful framework to operationalize the concept of social cohesion [17,18]. They suggested that social cohesion comprises two main dimensions. The first concerns the reduction of disparities, inequalities, and social exclusion. Examples are government efforts to provide equal employment opportunities for minorities. The second concerns strengthening social relations, interactions, and ties. They believe that a cohesive society needs to simultaneously consider both dimensions because a society only focusing on strong and intimate social relationships can lead to social exclusion. Berger-Schmitt and Noll also stressed that the availability and the quality of these relationships are both important [17,18].

There is a similarity between Bernard’s model and Berger-Schmitt and Noll’s conceptualization of social cohesion. Both emphasize social justice, social relationships, and social exclusion. Many of the recent debates on social cohesion revolve around issues of social exclusion and value diversity as well as appropriate responses to these issues [19]. The fundamental dilemma is how far social and value diversity is compatible with social cohesion and whether it may result in social exclusion such as excluding minority groups from certain job markets. Some researchers suggested that a high level of social cohesion entails a high level of social exclusion. They believe that a degree of cultural and value homogeneity is a necessity for a cohesive society [20]. In contrast, many other researchers with liberal attitudes argued that diversity is not a problem for social cohesiveness. They believe that in many Western societies, political institutions are sufficiently robust to mediate conflicting interests, and societies are well equipped with values of tolerance and respect for other cultures [6].

Wilkinson and Pickett’s well-known book “The spirit level: why more equal societies almost always do better” initiated debates and dialogues regarding the impact of income inequality and the function of social cohesion [7]. The book claimed that different health and social problems (i.e., mental health, drug use, obesity, teenage pregnancies et al.) were more prevalent in countries with higher degree of income inequality. The authors analyzed data in a sample of 23 developed countries and found that income inequality was associated with lower life expectancy, higher rates of infant mortality, higher prevalence of low birth weight, and higher rates of AIDS and depression. For instance, in Japan and Scandinavian countries where the income inequality was low, the life expectancy was significantly better than in U.S. and U.K. where the income inequality was high among developed countries. One of the proposed mechanisms was through social cohesion, implying that in unequal societies people become less likely to trust each other or to be involved in community life, and thus, the negative outcome on health [7].

### Regimes of social cohesion

While the above theories analyzed the theoretical dimensions and compositions of the concept of social cohesion, scholars recently suggested a contrasting approach of examining social cohesion by regimes [4,12,21]. The reason for using a typological approach is because countries or regions are not always homogeneous in possessing these dimensions of social cohesion (i.e., liberty, equality, and solidarity), but a diversity of countries with different combinations of these dimensions likely exist. There is a long tradition of understanding lasting differences between countries by regimes in which countries under the same regime are usually economically, socially, culturally, and sometimes geographically close to each other. For instance, Esping-Anderson identified regimes of welfare states in Western societies based on welfare capitalism [22].

Instead of identifying general dimensions of social cohesion applicable to all Western states, Green [4,21] and Janmaat [12] sought to empirically verify the unique and durable “regimes” of social cohesion. Green et al. [4] performed cluster analyses among 18 countries and found evidence for distinct English-speaking liberal and distinct Scandinavian social-democratic regimes, while little empirical support was found for the existence of a social market regime. To verify Green et al.’s results, Janmaat conducted cluster analyses among 16 countries and reported a reasonably distinctive and stable Scandinavian model characterized by high trust, high equality, and low crime rates. He also identified a continental European cluster, which exhibited unexpectedly low levels of social hierarchy and high levels of value pluralism and ethnic tolerance, but found no evidence of a distinctive liberal English-speaking regime of social cohesion [12].

Those studies generally reported that when a country is socioeconomically wealthy, it is usually more trusting, equal, safe, and tolerant toward minorities. Some studies suggested that liberal English-speaking countries had the strongest liberal attitudes and highest levels of individual freedom and thus had higher value diversity. However, some others observed that liberty is also strong in Scandinavia [4,12]. They thought high taxes and strong government intervention in Scandinavian countries form an egalitarian welfare state providing equal opportunities for different populations, which can foster liberal attitudes and value diversity. The key issue here might be the extent to which the relational and institutional systems, in particular democratic systems, are well structured and can provide equitable access and opportunities for people from different social groups [6].

### Empirical studies of social cohesion and health

How does social cohesion influence individual health? Some researchers suggested that social cohesion, underpinned by national policies and political decisions, may influence individual health through providing equal opportunity and mitigating poverty, disparity, and social exclusion [19]. For example, relevant policies provide opportunities for citizens to participate in social, economic, and political activities within communities, which would further enhance well-being. Social cohesion, manifested in policies that deliberately intervene unemployment, poverty, and health inequality, can also have positive effect on health through the re-allocation of social and health resources. A more cohesive society may invest more in public infrastructure such as education, social welfare, and health services, which narrow down health inequality and reduce unequal access to health services [23]. On the other hand, from the psychological and behavioral perspectives, social cohesion may exert an influence through social norms to reduce risky behavior and to diffuse health information. A higher level of social cohesion may also provide more social support and mutual respect, which can buffer the adverse effects of stress [24,25].

Some research, based on an ecological study design, looked at the relationship between national-level social cohesion and national-level health outcomes, such as mortality and morbidity. Some found strong effects of trust on mortality [26], while others found only modest or insignificant effects of trust and social participation on health [27,28]. Few studies used a multilevel study design to examine the impact of social cohesion on individual health above and beyond individual characteristics, but they produced inconsistent results. Mansyur [29] and Poortinga [30] showed that individual-level social participation and trust do not affect self-rated health; however, national-level social participation and trust do affect self-rated health. Using data from 69 countries, Jen and colleagues showed that social trust at both the national and individual levels was positively associated with self-rated health after controlling for individual sociodemographic and income variables [24].

Some other relevant studies were published from the aspect of welfare states [31]. Prior studies generally reached agreement that population health varies according to the type of welfare state regime, and a regime advocating more-egalitarian welfare policies (i.e., public medical services) is more likely to maintain and improve a nation’s health [32]. Most empirical studies identified Scandinavian welfare states as having the best population health status [33]. Although Asian welfare states had lower social expenditures compared to Western countries, studies found that they did not have higher infant mortality or lower life expectancy, probably because of a strong reliance on family to provide care [34,35]. Very few studies used a multilevel study design to examine the regimes of welfare states on individual health. Eikemo et al. [32] used a multilevel analytical approach and found that Scandinavian and Anglo-Saxon welfare regimes reported better self-rated health than Southern and Eastern European welfare regimes, after taking into account individual sociodemographic characteristics and social relationships.

This study intends to further understand the relationship between social cohesion and health. At present, most of the literature on social capital focused on small groups (i.e. neighborhoods) or used only two aspects, trust and social participation, to measure social cohesion. As we have emphasized, social cohesion should be characterized as features of a whole society incorporating attitudes, behaviors, institutional and structural dimensions that bound citizens together for better life quality. Therefore, we implemented a series of procedures to comprehensively measure and analyze the relationship between social cohesion and individual health. We first followed theoretical frameworks and identified multiple dimensions of social cohesion using a factor analysis. Then, we developed a typology of social cohesion regimes with a cluster analysis to further understand the spatial patterning of social cohesion in different countries. Finally, using a multi-level statistical design, we assessed the effects of dimensions of social cohesion on an individual’s self-rated health after controlling for individual characteristics.

## Methods

### Data

This study used a multilevel study design. At the individual level, study samples were from the 2000 World Value Survey (WVS) and the 2002 European Value Survey (EVS). The WVS and EVS are cross-nation surveys about social, economic, cultural, and political values and behaviors in different societies. Both surveys have almost identical questions, and are known to have a standardized study design and a rigorous data collection procedure. The WVS and EVS have executed fives waves of studies: 1981–1984, 1989–1993, 1994–1998, 1999–2004, and 2005–2008. Stratified random sampling was conducted to represent population in each society. Data collection methods included face-to-face interviews or phone interviews for remote areas. Extensive descriptions of the WVS and EVS can be found at the respective websites (http://www.worldvaluessurvey.org and http://www.europeansocialsurvey.org).

This study used individual-level samples from 29 high-income countries, since theories on social cohesion and welfare states were originally constructed for understanding the influences of social, political and economic factors in these developed countries. Prior relevant studies focused on these high-income countries, as well. For less developed countries, other theories, such as dependency theories, may be more suitable to be used as the framework to guide studies of this nature [36]. Data on Austria, Belgium, Czech Republic, Denmark, Finland, France, Germany, Greece, Hungary, Ireland, Italy, Luxembourg, Netherlands, Poland, Portugal, Slovenia, Sweden, and United Kingdom were derived from EVS. Data on Australia, Canada, Hong Kong, Japan, Korea, New Zealand, Norway, Spain, Switzerland, Taiwan, and United States were derived from WVS. National-level social cohesion statistics were obtained from Organization of Economic Cooperation and Development (OECD) datasets, World Development Indicators (WDI), Asian Development Bank (ADB) key indicators for the year 2000, and from aggregating responses from the WVS. We chose the time period around 2000 because a more-comprehensive list of variables and data from more countries were collected at the millennium. In total 47,923 individuals were included in this study (Table 1).

**Table 1:** Sample description

**Country**	**Number of individuals**	**Percent good health**
Australia	2047	79.38
Austria	2239	75.35
Belgium	1782	78.23
Canada	1921	81.10
Czech Republic	1263	49.56
Denmark	1473	78.41
Finland	1991	66.85
France	1501	60.36
Germany	2895	58.58
Greece	2560	71.09
Hong Kong	1225	63.43
Hungary	1629	44.94
Ireland	2037	83.60
Italy	1205	63.90
Japan	1341	54.74
Korea	1200	77.50
Luxembourg	1436	64.00
Netherlands	2359	73.12
New Zealand	1175	78.98
Norway	1127	79.24
Poland	2089	55.29
Portugal	1510	46.75
Slovenia	1508	56.50
Spain	1206	74.54
Sweden	1994	73.22
Switzerland	1203	83.13
Taiwan	780	47.56
United Kingdom	2028	70.96
United States	1199	83.49

### Measurement

#### National-level characteristics

We adapted Berger-Schmitt and Noll’s framework to measure social cohesion [17,18] and also considered Bernard’s conceptualization [3]. Berger-Schmitt and Noll’s framework included a comprehensive list of well-grounded indicators for measuring the concept of social cohesion, comprising multiple dimensions such as equality, individual freedom, social welfare, trust to individuals and institutes, and state power [6]. In particular, the framework has carefully considered how to incorporate social exclusion in the measurements. Compared to prior theoretical literatures, Berger-Schmitt and Noll’s framework has made a major contribution to the operationalization of social cohesion [6].

Berger-Schmitt and Noll’s framework of social cohesion suggests several dimensions: social and political attitudes, social exclusion, equal opportunities, regional disparities, the availability of social relations, the quality of social relations, and the quality of social institutions. Table 2 shows the operational definitions and statistics of the variables.

**Table 2:** Measurements and descriptive statistics for the variables analyzed

**Dimensions**	**Indicators**	**Questions and items**	**Operation definition**	**Mean**	**Min, max**	**Percentage**	**Data source**
**Country-level (N = 29)**							
Social and political attitudes							
	Liberty aspiration	If you had to choose, which one would you say is most important? And which would be the next important?	Respondents’ priorities were “giving people more say in important government decisions” and “protecting freedom of speech”. Assigning 3 points for both items on first and second rank, 2 points for one of these items on first rank, 1 point for one of these items on second rank, and 0 for none of these items on first or second rank. The mean of the aggregate score was computed.	1.34	0.71, 1.92	---	WVS/EVS
		1. Maintaining order in the nation					
		2. Giving people more say in important government decisions					
		3. Fighting rising prices					
		4. Protecting freedom of speech					
	Democratic attitude	Could you please tell me if you agree with the following items at a 4-point scale?	The index was created by adding up the four items. The higher the value, the more-positive attitude toward democracy. The direction of item 1, 2, and 3 were reversed. Mean of the sum of scores of these four items was computed.	11.49	9.82, 12.71	---	WVS/EVS
		1. In democracy, the economic system runs badly					
		2. Democracies are indecisive and have too much quibbling					
		3. Democracies aren’t good at maintaining order					
		4. Democracy may have problems but it’s better than any other form of government					
	Value diversity	Please tell me whether you think it can always be justified, never be justified or something in between along a 10-point scale.	The sum of the standard deviation of each statement was calculated	14.21	11.92, 16.69	---	WVS/EVS
		1. Homosexuality					
		2. Abortion					
		3. Divorce					
		4. Euthanasia					
		5. Suicide					
Social exclusion	Ethnic tolerance	Could you please sort out any that you would not like to have as neighbors?	The percentage of not mentioning immigrants	79.04	6.21, 97.40	---	WVS/EVS
	Immigrant percentage		The percentage of immigrants by total population	10.37	1.30, 39.40	---	OECD, ADB
Equal opportunities	Gender employment ratio		Female to male employment ratio	0.76	0.56, 0.94	---	OECD, ADB
	Gender wage gap		The difference between male and female earnings expressed as a percentage of male earnings	18.47	7.10, 40.40	---	OECD, ADB
	Social expenditure		Percentage of GDP	19.96	5.26, 30.10	---	OECD, ADB
	Health expenditure		Percentage of GDP	7.48	4.89, 13.00	---	OECD, ADB
	Physician density		Number of physicians per 1,000 people	2.71	1.16, 4.23	---	OECD, ADB
	Education expenditure		Percentage of GDP	5.23	2.80, 8.10	---	WDI, ADB
	Government responsibility	How would you place your views on this scale?	The percentage of people who placed their view as ≤ 5 was determined	56.52	13.92, 79.25	---	WVS/EVS
		The government should take more responsibility to ensure that everyone is provided for versus people should take the responsibility to provide for themselves.					
		The responses exhibited a range from 1–10, with 1 being “government should take more responsibility” and 10 being “people should take more responsibility”.					
Regional disparity	Gini index × 100		Gini coefficients of family income	31.67	22.50, 52.50	---	WDI, ADB
Availability of social relations	Association membership		Percentage of people who had joined at least one among a list of 15 voluntary organizations	60.04	14.37, 95.66	---	WVS/EVS
Quality of social relations	Social trust	Generally speaking, would you say that most people can be trusted or that you need to be very careful in dealing with people?	Percentage of people reporting 1	36.73	12.30, 66.53		WVS/EVS
		1. Most people can be trusted					
		2. Need to be very careful					
Quality of social institutions	Trust in civil service	How much confidence you have in the civil service? A great deal, quite a lot, not very much, or none at all	Percentage of “a great deal” and “quite a lot”	44.42	14.34, 66.58	---	WVS/EVS
**Individual-level (N = 47,923)**							
	Self-rated health (good health)		Fair and bad health (reference) vs. good and very good health	---	---	68.43	WVS, EVS
	Gender (men)		Women (reference) vs. men	---	---	46.97	WVS, EVS
	Age		Age in years	45.96	14, 110	---	WVS, EVS
	Educational level		Primary education (reference)	---	---	27.71	WVS, EVS
			Upper secondary education			52.06	
			Tertiary education			20.23	
	Income		Lower tertile (reference)	---	---	26.19	WVS, EVS
			Middle tertile			35.54	
			Upper tertile			38.27	

ADB, Asian Development Bank; EVS, European Value Survey; OECD, Organization of Economic Cooperation and Development; WDI, World Development Indicators; WVS, World Value Survey.

In terms of social and political attitudes, liberty aspirations, democratic attitudes, and value diversity were used as indicators. Liberty aspirations represent the level of emphasis on freedom of expression and political participation relative to material safety. This concept, developed by Welzel and Ingelharts, was suggested to have a great impact on the democratization of a country [37,38]. Democratic attitudes described whether there was public support for democratic principles and systems. Value diversity represented the tolerance of a society toward social diversity and was measured as the dispersion of opinions on several social issues [4]. We used ethnic tolerance and immigrant percentage to represent the dimension of social exclusion. Ethnic tolerance described whether participants dislike having immigrants as neighbors. The position on immigrant members of a society was suggested to be the most sensitive indicator for assessing social exclusion [39]. Immigrant percentage was measured as the percentage of immigrants by total population in a country. We used several items to represent the dimension of equal opportunities. The ratio of female/male employment rates and gender wage gap represented whether a country had equal employment opportunities for women and men. Social expenditures, educational, and health expenditures as well as physician density measured the government efforts and investment to increase equality. Government responsibility showed people’s attitudes toward government versus individual responsibility in providing social services [40]. For regional disparities, we used Gini coefficients to measure inequalities of household incomes. For availability of social relations, we measured membership in associations by asking respondents to indicate their membership in clubs and associations. We used social trust to represent the quality of social relations [41]. Last, we used trust in the civil service to measure the quality of social institutions [41].

#### Individual-level characteristics

Self-rated health, measured by the identical questions in WVS and EVS: “How would you rate your general state of health?”, was rated as “poor”, “fair”, “good”, or “very good”. It was recoded as a dichotomized variable with “poor”, and “fair” as 0 to represent poor health and “good”, and “very good” as 1 to represent good health.

Age, gender, educational level, and household income were included as control variables in the multilevel models. Educational level was measured by asking respondents their highest level of education achieved and ranged from 'did not complete primary education’ to 'second stage of tertiary education’, and was regrouped into three categories: primary(lower), upper secondary(middle), and tertiary education(upper). Income was measured by the annual household income. Individuals were then classified into lower, middle, and upper levels of income.

### Analysis

An exploratory factor analysis was conducted to identify dimensions of social cohesion. The exploratory factor analysis seeks to discover if the observed variables can be explained largely in terms of a much-smaller number of latent factors, and to further uncover the interrelationships among the observed variables and the underlying theoretical factors [42]. Each factor was defined as a linear combination of optimally weighted observed variables. In this study, participants’ scores on the questionnaires items would be weighted and then summed to compute their scores on each factor (dimension) of social cohesion. The method of extraction was a principal component analysis with an oblique (promax) rotation procedure since domains of social cohesion were reported to be correlated [12]. The number of factors was decided by a significant jump in the slope of a scree plot and an eigenvalue of > 1, and the ability to interpret different factor solutions.

A cluster analysis with Ward’s algorithm was used to identify a typology of regimes based on dimensions generated by the exploratory factor analysis. Cluster analysis was primarily developed for classifying cases into relatively homogeneous groups and creates a different regimes based on selected variables [43]. To determine the number of clusters, an inverse scree plot was used. A significant jump in the fusion coefficient in the inverse scree plot was used to inform the number of clusters extracted from the data [43]. We also used statistics of the cubic clustering criterion (CCC), pseudo F (PSF), and pseudo t^2^ (PST2) to verify the number of clusters.

Multilevel regression models were applied to assess the influences of social cohesion on an individual’s self-rated health. We used SAS GLIMMIX to fit multilevel models with a binomial distribution assumption and a logit link [44]. The estimation method was a restricted maximum-likelihood procedure. Model one was first fitted with only intercept to access the ICC of the intercept-only model. Model two was fitted with individual sociodemographic characteristics. Model 3 was fitted with social cohesion dimensions. Individual sociodemographic characteristics were added in Model 4 to assess the effects of social cohesion dimensions after controlling for individual-level characteristics.

We also calculated intra-class correlation (ICC) in each model, which represents the proportion of variance at the group level divided by the sum of the variances at the individual level and the group level. According to Snijders and Bosker [45], for binary outcome, the unobserved individual variance follows a logistic distribution with individual level variance V_i_ equal to π^2^/3 (that is, 3.29). Therefore the ICC for binary outcomes is calculated as follows with V_A_ representing variance between groups (countries).

$$\text{ICC}=V_{A}/\left(V_{A}+3.29\right)$$

We used the likelihood ratio test to compare different models. The positive difference of -2 log likelihoods between two models has a Chi-squared distribution with the degrees of freedom obtained from the difference of the number of parameters in the two models [45]. If the Chi-squared test is significant, this suggests a more parsimony model with fewer parameters is preferred.

## Results

### Dimensions of social cohesion

Table 3 shows the five factors extracted from the exploratory factor analysis. Social expenditure, physician density, gender wage gap, and trust in civic services formed the first factor (alpha = 0.80). This factor can be referred to as the social equality factor. Items were related in different direction: the higher social expenditure and physician density, the lower gender wage gap. Interestingly, countries with higher social expenditure tended to have lower trust in civic services, which was probably due to a number of Eastern European countries included in our analysis. After the collapse of communism in 1989, these countries had high scores on physician density and social expenditure, but had relatively low scores on trust in civic services. Government responsibility, health spending, ethnic tolerance, and value diversity combined to form the second factor (alpha = 0.75). This factor can be referred to as the social inclusion factor. Items suggested that when a country had higher tolerance toward immigrants, it also had higher value diversity, and higher government efforts’ to ensure that everyone is provided. This factor also indicated these countries were more likely to have higher health expenditure [46]. Education expenditures and gender employment ratio formed another factor, and can be referred as the social development factor (r = 0.60), representing the level of investment in human capital and in gender equity. The fourth factor included social trust, democratic attitude, and social participation (alpha = 0.78), and can be referred to as the social capital factor. The democratic attitude was highly loaded with the other two traditional social capital items, trust and social participation, suggesting that democratic attitude could be a key element of social capital. The last factor, referred to as social diversity factor (alpha = 0.57), comprised items of percentages of immigrants, Gini index, and liberty aspiration. This factor showed that countries with a higher percentage of immigrants tended to have a higher Gini coefficient, but also have a higher level of liberal attitude. Inter-factor correlations among the five factors ranged from 0.004 to 0.37.

**Table 3:** Factor analysis of social cohesion characteristics

	**Social equality**	**Social inclusion**	**Social development**	**Social capital**	**Social diversity**
Physician density	0.73^*^	0.23	-0.23	0.04	-0.12
Social expenditure	0.71	0.23	0.24	-0.07	-0.16
Gender wage gap	-0.75	0.01	-0.3	0.35	-0.24
Trust in civic service	-0.84	0.23	0.27	-0.10	0.16
Government responsibility	-0.10	0.83	0.23	-0.09	-0.02
Health spending	0.01	0.71	0.08	0.09	0.03
Ethnic tolerance	0.08	0.67	0.08	0.10	0.06
Value diversity	0.34	0.40	0.01	0.19	0.06
Educational expenditure	-0.02	-0.01	0.85	0.17	-0.08
Gender employment ratio	-0.10	0.16	0.81	-0.01	-0.04
Social trust	0.01	-0.31	0.48	0.78	0.06
Democratic attitude	-0.08	0.29	-0.19	0.72	-0.16
Membership in association	-0.14	0.18	0.29	0.71	0.02
Immigrant percentage	-0.10	0.05	0.03	0.02	0.88
Gini index	-0.24	-0.06	-0.35	-0.20	0.68
Liberty aspirations	0.33	0.20	-0.10	0.49	0.55

^*^ Underlined factor loadings indicated variables of the same factor.

### Regime of social cohesion

A cluster analysis was carried out using the dimensions of social cohesion identified by the factor analysis. Results of the scree plot, and CCC, PSF, and PST2 statistics all point to six potential clusters of social cohesion states. Table 4 listed countries and the mean scores, as well as rankings, of the dimensions of social cohesion by clusters. Cluster I, consisted of East Asian countries, ranked low in most dimensions, except in social diversity. Cluster II, consisted of countries in Southern Europe, ranked high in social equality but low in social inclusion. Cluster III, consisted mainly of countries in Central and Eastern Europe and a few West European countries, had low ranking in social capital and social diversity. Cluster IV, consisted of Scandinavian countries, ranked high in social equality, social development, but low in social diversity. Cluster V, consisted of Netherlands, Germany, and Austria, ranked high in social inclusion and social capital, but low in social development. Cluster IV, consisted mainly of countries in North America and Australasia, and New Zealand, ranked high in social inclusion, social development, and social diversity, but low in social equality.

**Table 4:** Mean scores and rankings of dimensions of social cohesion by clusters

	**Cluster I**^**a**^		**Cluster II**^**b**^		**Cluster III**^**c**^		**Cluster IV**^**d**^		**Cluster V**^**e**^		**Cluster VI**^**f**^	
**Dimensions**	**Score**	**Rank**^**g**^	**Score**	**Rank**	**Score**	**Rank**	**Score**	**Rank**	**Score**	**Rank**	**Score**	**Rank**
Social equality	-1.71	L	1.17	H	0.17	M	0.39	H	0.36	M	-0.15	L
Social inclusion	-1.81	L	-0.23	L	0.02	M	0.29	M	0.95	H	0.61	H
Social development	-0.76	L	-0.15	M	0.09	M	1.73	H	-0.27	L	0.13	H
Social capital	-1.73	L	0.02	M	-1.04	L	1.13	H	0.98	H	0.43	M
Social diversity	0.03	H	-0.13	M	-0.43	L	-0.49	L	-0.31	M	1.17	H

^a^Hong Kong, Japan, Korea, and Taiwan;

^b^Greece, Italy, and Spain;

^c^Belgium, Czech Republic, France, Hungary, Ireland, Poland, Portugal, Slovenia, and United Kingdom;

^d^Denmark, Finland, Norway, and Sweden;

^e^Netherlands, Germany, and Austria;

^f^Australia, Canada, Luxembourg, New Zealand, Switzerland, and United States;

^g^Ranking of H indicating the dimension score ranked in the top two; M indicating the dimension score ranked in the middle two; L indicating the dimension score ranked in the lowest two among the six clusters.

### Multilevel analyses of effects of social cohesion on self-rated health

Table 5 shows the results of the multilevel analysis. Model 1 was the intercept-only model, while Model 2 included only individual-level characteristics. Individuals with certain characteristics, such as being male, younger, having a higher or middle educational level, and having a higher or middle income, reported better health than their counterparts.

**Table 5:** Effects of dimensions of social cohesion on individual self-rated health

	**Model 1**	**Model 2**	**Model 3**	**Model 4**
Intercept estimate	0.80	1.90	0.80	1.90
**Social cohesion dimensions**				
Social equality			-0.12	-0.02
			(0.08)^a^	(0.09)
Social inclusion			0.178*	0.21*
			(0.08)	(0.09)
Social development			-0.01	-0.05
			(0.07)	(0.08)
Social capital			0.30*	0.28*
			(0.07)	(0.08)
Social diversity			0.20*	0.22*
			(0.07)	(0.08)
**Individual variables**				
Gender (male/female)		0.23*		0.23*
		(0.02)		(0.02)
Age		-0.03*		-0.03*
		(0.01)		(0.01)
Educational level (high/low)		0.64*		0.64*
		(0.03)		(0.03)
Educational level (middle/low)		0.40*		0.40*
		(0.03)		(0.03)
Income (high/low)		0.47*		0.47*
		(0.03)		(0.03)
Income (middle/low)		0.18*		0.18*
		(0.02)		(0.02)
ICC	0.08	0.09	0.04	0.05

^*^p < 0.05.

^a^Standard error presented in parenthesis.

Model 3 and Model 4 assessed the relationship between dimensions of social cohesion and self-rated health. Three dimensions of social cohesion, including social inclusion, social capital, and social diversity, were significantly associated with individual-level self-rated health before and after controlling individual sociodemographic characteristics, as indicated in Model 4.

Based on the formula provided by Snijders and Bosker [45], the ICC is 0.08 for the intercept-only model. The residual ICC became 0.09 after adding individual-level characteristics. With social cohesion dimensions, the residual ICC was 0.04 and became 0.05 with the addition of individual-level variables in Model 4. Compared to Model 1, the smaller ICC in Model 3–4 and the significant likelihood ratio tests indicated that the addition of the social cohesion variables has led to a significant reduction in unexplained variance between countries.

## Discussion

In this study, the five distinct dimensions of social equality, social inclusion, social development, social capital, and social diversity, partly confirmed Bernard’s model of democratic dialogue, which included liberty, equality, and solidarity [3]. Three of the five dimensions, social inclusion, social capital, as well as social diversity, were significantly associated with individual health. This study demonstrated that people in countries with higher social inclusion were more likely to report better health even after controlling for other individual characteristics. Similar results have been revealed from prior research focusing on the effect of social exclusion on health [47]. Although social inclusion and social exclusion are not exactly opposite of each other, our finding does show consistent direction of effects. Social inclusion may affect individual health through participation in economic, social, and political activities. Furthermore, social inclusion may also influence individual social position and social class, which in turn may affect one’s health through increased access to resources that are beneficial to health, as well as enhancing the development of equitable social relationships. A society that values diversity and includes all citizens in welfare schemes may also likely foster an environment that allows individuals to have more locus of control in access to health and social services, which may also have a positive effect on self-rated health. This finding suggests that in developing a social welfare or a health care system, government should consider not only the universality of coverage, but probably should also consider the special needs of subgroups within the population.

This study once again demonstrated that social capital, which represents the principal of solidarity, is a crucial dimension of social cohesion, and the influence of social capital on individual health should not be overlooked. Social capital, in this study, was comprised of social trust, social participation, and democratic attitude. Both cognitive aspect (i.e. social trust) and structural aspect (i.e. social participation) of social capital had beneficial impact on self-rated health. Consistent with prior studies, Pooartina [30] and Mansyur [29] showed that country-level social participation and trust were positively associated with self-rated health. Jen and colleagues [24] also showed that social trust at the national-level was positively associated with self-rated health above and beyond individual socio-demographic characteristics in a sample of 69 countries. Previous literature and finding from this research point to the direction that promoting an social environment that are favorable for involvement in social membership, as well as nurturing public trust in government, may improve self-rated health, and thus, probably should be considered a policy priority.

In this study, the dimension of social diversity was positively related to individual health after controlling for individual characteristics. The dimension of social diversity suggested that when a country had a higher score on liberal aspiration, it also had a higher percentage of immigrants, as well as a higher score on Gini index, which implied a greater disparity in income. Since the dimension of social diversity consists of Gini index of family income, the results of the positive relationship between social diversity and self-rated health are contradictory to the impression that countries with higher income inequality have worse population health. One main reason is that the dimension of social diversity also measures other social conditions such as immigrant concentration and liberal attitude, and thus masked the true effect of Gini index. It is also possible that countries with a higher percentage of immigrants were countries with a high demand for labor, and thus had an open immigration policy. Immigrants, in general, would tend to have lower income, yet more likely to be younger and therefore may perceive themselves to be in better health. Thus, a high percentage of younger and lower income immigrants may offset the true effect of Gini index on health. The other explanations may be differences in the outcome variables used, the level of the statistical analysis, and number of countries selected. Rather than using individual self-rated health as the outcome variable, most of the studies reviewed by Wilkinson and Pickett regarding income inequality and health conducted ecological analyses with group-level outcomes (i.e., mortality, life expectancy) and did not control individual-level sociodemographic factors. In addition, those studies focused on 23 richest countries as the target sample, and more societies from East Europe and East Asia were included in this study.

The positive relationship between social diversity and health can probably be explained from the perspective of health policy. It is possible that countries with a high social diversity are more likely to formulate health policies that respect individual freedoms and differences. Hence such societies may provide an environment more conducive for individuals to pursue health, and be more tolerant of conditions that may be associated with deviant behaviors, particularly mental or psychological conditions. In this study, the item of liberty aspiration reflected only social attitudes and norms toward individual liberty and freedom [3]. Future research should attempt to incorporate variables such as care utilization, open markets, and entrepreneurial liberty if such information is available.

Our findings should be considered in light of the following limitations. First, we did not examine longitudinal social cohesion measurements, which may have generated selection bias [48]. Although we did control a number of important individual sociodemographic characteristics, the relationship between social cohesion and individual self-rated health could be due to some unmeasured individual characteristics, which would lead to biased estimates of social cohesion effects. In addition, individuals with better health status are more likely to participate in civic activities and perceive the environment as a more trustful and safer place; therefore, the relationship found between social cohesion and self-rated health should perhaps be more cautiously interpreted as correlations rather than as direct evidence of the influences of social cohesion. Second, the study did not measure all aspects of social cohesion. However, compared to prior studies, we measured more aspects of social cohesion including liberal aspirations, value diversity, democratic support, and government interventions in inequality. Future research needs to improve measurements by including additional aspects of social cohesion. Although we did not directly assess the validity of the measurements, our measures exhibit content validity by covering each domain of Berger-Schmitt and Noll’s framework [17,18]. Many items used in our studies were found to have significant relationships with other theoretical concepts and health outcomes in previous studies and thus exhibit construct validity [29,30]. For example, the items of social trust, membership in associations, and welfare states were positively correlated with mortality, self-rated health, and other health behaviors [26-30]. Third, the outcome measure, self-rated health, is more likely to be influenced by social and cultural norms, which might not be appropriate for use in Asian societies [32]. Asians do not exhibit extreme expressions in social interactions, they tend to give mid-point responses rather than express definite agreement or disagreement when they are asked about their health status [49]. Therefore Asian countries would score lower in self-rated health according to the way we lumped fair and poor health in the same category. Because of the aforementioned debates, some studies suggested that self-rated health is not an appropriate assessment for cross-nation comparison on individual health status [50]. Nevertheless, others suggested self-rated health is a good predictor of mortality in different countries [51]. However, our multilevel statistical analysis should explain at least some parts of the variance between countries in the measurement of self-rated health due to cultural, historical, and institutional factors [52]. Fourth, using national averages to measure social cohesion can hide important variations within countries concerning the attitudes of interest. It is thus possible that countries with similar mean values for some attitudes have different variations. Last, missing data are also a problem because of the available datasets. We limited our analyses to those countries for which there were no missing data for all of our predictors. This strategy may overlook the most disadvantaged populations, who were less likely to participant in the survey studies [53].

## Conclusions

Limitations aside, this study is an innovative effort to incorporate different aspects of social cohesion. This study suggests that some dimensions of social cohesion, such as social inclusion, social capital, and social diversity, were associated with individual self-rated health even after controlling for individual characteristics. Findings from this research may partially explain why some countries have similar income, service provision, and resources, but have different population health outcome. While service utilization and resource allocation do have significant impact on population health, governments, especially those of developed countries, should also develop policies to foster a society with a high level of social inclusion, social capital, and social diversity, to achieve further advancement in population health. Future research could focus on identifying possible pathways by which social cohesion influences various health outcomes.

## Competing interests

The authors declare that they have no competing interests.

## Authors’ contributions

YCC participated in study design, data analysis and writing the manuscript. KYC participated in study design and writing the manuscript. THY participated in data collection and data analysis. All authors read and approved the final manuscript.
